# The actin-bundling protein fascin is overexpressed in colorectal adenomas and promotes motility in adenoma cells *in vitro*

**DOI:** 10.1038/sj.bjc.6605286

**Published:** 2009-09-08

**Authors:** D Qualtrough, K Singh, N Banu, C Paraskeva, M Pignatelli

**Affiliations:** 1 Department of Cellular and Molecular Medicine, School of Medical Sciences, University of Bristol, University Walk, Bristol BS8 1TD, UK

**Keywords:** colon, adenoma, fascin, migration

## Abstract

**Background:**

Fascin is overexpressed in many cancers, including colorectal, but its role in the malignant transformation of benign colorectal adenomas is unclear.

**Methods:**

Immunohistochemical analysis of fascin expression was carried out in resected human colorectal adenoma specimens. The effects of forced overexpression of fascin on adenoma cell motility were also analysed.

**Results:**

We show fascin overexpression in adenomas increasing with tumour size, histological type, and degree of dysplasia and increased cell motility in adenoma cell lines following fascin transfection.

**Conclusion:**

These data suggest an important role for fascin in the malignant progression of colorectal tumours.

Colorectal cancer development is widely accepted to involve the multifaceted progression of a benign lesion (adenoma) to invasive adenocarcinoma (hereafter referred to as ‘carcinoma’) by acquisition of a migratory, and ultimately invasive phenotype (Muto *et al*, 1975). The precise nature of the changes required for the malignant progression of adenomas remain poorly understood but prognosis is widely accepted to correlate with both the physical size and histological type of the adenomatous polyp, as well as the degree of cellular dysplasia displayed by the tumour cells (Muto *et al*, 1975).

The changes in motile behaviour demonstrated by tumour cells require dynamic rearrangements in the actin cytoskeleton, which are governed by multiple actin-binding proteins (Matsudaira, 1994). The 55 kDa fascin-1 protein (hereafter be referred to simply as fascin) localises to the core actin bundles of spikes and filopodia at the leading edge of migratory cells, and has been shown to increase migration in several cell types (Adams, 2001; Kureishy *et al*, 2002). There now exists a plethora of reports showing a general trend across epithelial tissues with fascin being absent from normal epithelium and present in tumours of the same tissue origin (Hashimoto *et al*, 2005).

We have previously shown that fascin is absent from normal colorectal epithelium but overexpressed in colorectal carcinomas (Sawhari *et al*, 2003). Two recent studies have demonstrated that fascin expression correlates with a poor prognosis in colorectal cancers (Hashimoto *et al*, 2006; Puppa *et al*, 2007). Hashimoto *et al*. (2006) found no correlation between the genetic origin of colorectal adenomas (FAP or sporadic) and fascin expression (Hashimoto *et al*, 2006). However, to date, no study has been reported analysing the expression and distribution of fascin in benign colorectal adenomas in relation to the clinical risk factors associated with malignant tumour progression.

To understand the role of a gene during tumourigenesis, it is important to know at what stage it becomes deregulated. In the study presented here, we sought to determine this potential relationship between fascin expression and malignant progression in colorectal adenomas. We performed immunohistochemistry for fascin on colorectal adenoma specimens graded for the risk factors associated with malignant potential, namely polyp size, histological type and the degree of dysplasia. To corroborate these findings we studied fascin expression in colorectal adenoma-derived cell lines and determined the effect of fascin overexpression on cell motility in these non-malignant cells.

## Materials and methods

### Immunohistochemistry

Anonymised, archival, paraffin-embedded material was obtained from the files of the Department of Histopathology, Bristol Royal Infirmary following ethical approval (04/Q2003/49). A total of 64 surgically resected colorectal adenomas were categorised on the basis of polyp size, histological type and the degree of dysplasia as outlined in Table 1. We also examined 10 samples of normal colorectal mucosa.

Fascin was detected using a mouse monoclonal antibody (Dako-Cytomation, Glostrup, Denmark) as previously described (Jawhari *et al*, 2003). Negative controls had no primary antibody applied.

Stained samples were scored by two independent observers (DQ and NB) in terms of the proportion of epithelial cells staining positive (1 for <20%, 2 for 20–80% and 3 for >80%) and also the intensity of epithelial staining relative to that observed in adjacent endothelial cells (0, 1, 2, 3 for negative, weak, moderate and strong, respectively). The adenomas were also scored for focal fascin intensity around the tumour stalk.

### Cell lines

The adenoma cell lines AA/C1, AN/C1, BH/C1 and RG/C2 have been described previously (Paraskeva *et al*, 1984, 1989). All of the adenoma-derived cell lines are anchorage-dependent and are non-tumorigenic in athymic-nude mice (Paraskeva *et al*, 1989). HT29 and SW480 were derived from sporadic colonic adenocarcinomas (Fogh and Trempe, 1975; Leibovitz *et al*, 1976).

### Western blot analysis

Samples of 2 × 10^6^ cells were prepared for western blotting as described by Qualtrough *et al* (2007). A mouse monoclonal antibody raised against fascin was obtained from Dako (Dako-Cytomation, Carpinteria, CA, USA). Blots were subsequently probed with anti-*α*-tubulin (Sigma, Poole, UK) to show equal sample loading.

### Transfection and *in vitro* migration assays

For fascin overexpression studies, cells were seeded in triplicate in 25 cm^2^ flasks and grown to 90% confluency. Cells from each cell line were then transfected with either 10 *μ*g pcDNA3 (Invitrogen, Paisley, UK) or 10 *μ*g pcDNA3/human fascin-1 (Jawhari *et al*, 2003) using Transfast (Promega, Southampton, UK) following the manufacturers instructions, or medium changed as untransfected controls. The pcDNA3/human fascin-1 plasmid was a kind gift from Dr Jo Adams (University of Bristol). After 24 h transfection, the cells were prepared for motility assays with a sample being simultaneously prepared for western blotting to confirm the efficacy of transfection.

Cell migration assays were carried out using a transwell filter migration assay as previously described (Qualtrough *et al*, 2007). In this instance, the lower chamber was filled with calcium free-DMEM supplemented with 5% FBS to act as an attractant. After a 24 h incubation and staining with haematoxylin, cells on the lower filter surface were considered migratory and counted in 10 fields at × 20 magnification.

## Results

### Fascin expression correlates with adenoma size, histological type and the degree of dysplasia, and is largely focussed around the polyp stalk

**Figure 1:**
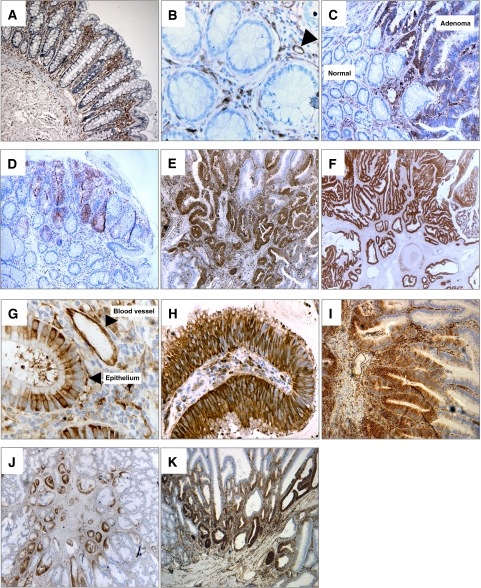
Fascin is overexpressed in colorectal adenomas: immunoreactivity correlates with tumour size, histology, and dysplasia and is focussed towards the stalk. Fascin immunoreactivity was detected in specimens of human colorectal adenomas by immunoperoxidase staining. (**A** and **B**) Normal colorectal mucosa showing positive staining in the stroma (arrowheads indicates endothelial cells in **B**), × 100 and × 400 magnifications, respectively. (**C**) The contrast in fascin immunoreactivity between a large tubulovillous adenoma with severe dysplasia and the adjacent non-neoplastic tissue (labelled), × 100 magnification. (**D**, **E** and **F**) Representative figures showing fascin immunoreactivity in a small tubular (**D**, × 100), medium tubulovillous (**E**, × 50) and large tubulovillous (**F**, × 25) adenoma. (**G** and **H**) Higher magnification images to show the subcellular distribution of fascin immunoreactivity within the adenoma epithelium: **G**, a medium tubulovillous adenoma with mild dyspplasia, × 400; **H**, medium tubular adenoma with moderate dysplasia, × 400 (arrowheads, endothelial cells in **G**). (**I**, **J** and **K**) Images showing the focal localisation of fascin expression around the adenoma stalk (**I**, medium tubulovillous adenoma, × 100; **J**, medium tubular adenoma, × 25; **K**, large tubulovillous adenoma, × 50).

Fascin immunohistochemistry was performed on 10 samples of normal colorectal mucosa and 64 colorectal adenomas categorised as shown in Table 1. Figure 1A and B show fascin immunoreactivity in normal colonic mucosa in longitudinal and transverse sections of the crypt-cuff axis, respectively. Positive staining was observed in the lamina propria, within endothelial cells (arrowed in Figure 1B and also Figure 1G), fibroblasts, and infiltrating lymphocytes. The epithelial tissue was uniformly negative for fascin.

Immunohistochemical analysis of colorectal adenomas showed that fascin expression was upregulated in the tumour epithelium and correlated with tumour size, tubulovillous histology and the degree of dysplasia. Figure 1C demonstrates the difference in fascin immunoreactivity between normal epithelium and adenomatous cells.

Of the 64 adenomas examined, 47 exhibited positive epithelial fascin immunoreactivity representing 43% of small (<0.5 cm), 77% of medium (05–3.5 cm) and 90% of large (>3.5 cm) adenomas. Statistical analysis of the data shows a significant correlation between polyp size and both the proportion of positive epithelial cells and the intensity of fascin immunoreactivity (scored relative to the endothelium), see Table 2. Figure 1D, E and F show representative examples of fascin staining in small, medium and large adenomas, respectively. These pictures clearly show the increasing proportion of positively stained epithelial cells and intensity of fascin staining in relation to polyp size.

Observation of the adenomas at higher magnification showed a cytoplasmic subcellular distribution as shown in Figure 1G and H with no nuclear staining. In Figure 1G, the strongly positive endothelial cells of a blood vessel can be clearly seen (arrowed) adjacent to an epithelial gland showing mild dysplasia and cytoplasmic fascin. Figure 1H shows a moderately dysplastic epithelial gland with fascin immunoreactivity in the cytoplasm but more intense than the mildly dysplastic adenoma shown in Figure 1G. Statistically across the sample group, the proportion of fascin-positive tumour epithelium and staining intensity correlated significantly (Table 2) with increasing dysplasia (mild, moderate or severe) exhibited by the adenoma (Figure 1G and H).

The adenoma samples studied were also graded according to histological type (tubular or tubulovillous) with a villous component observed in 17, 64 and 100% of small, medium and large adenomas, respectively (Table 1). Positive fascin immunoreactvity was found in 80% of tubulovillous adenomas (Figure 1F and K) compared with 55% in the tubular group (Figure 1D and J). Only 5% of tubular adenomas contained more than 20% fascin-positive epithelial cells compared with 34% of the tubulovillous group.

The most intense epithelial staining for fascin, particularly in the medium and large adenomas, tended to be focussed towards the stalk of the polyp (Figure 1I, J and K). Only 14% of small adenomas displayed this localisation, whereas 50% of medium and 85% of large adenomas showed focal staining towards the tumour stalk and this trend with polyp size was found to be statistically significant (Table 2).

**Figure 2:**
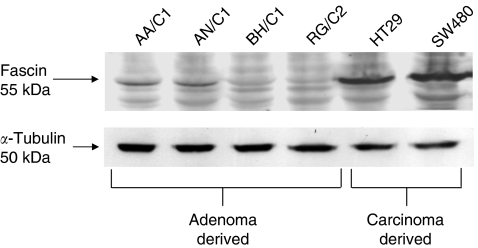
Fascin is more highly expressed in colorectal carcinoma cell lines compared with those derived from adenomas. Western blot analysis of fascin expression in cell lines derived from colorectal adenomas (AA/C1; AN/C1; BH/C1; and RG/C2) and carcinomas (HT29 and SW480). This blot is representative of three independent experiments. The blot was reprobed for *α*-tubulin to show even sample loading.

Western blotting was used to assess the native levels of fascin expression in adenoma-derived cell lines compared with widely used carcinoma-derived cell lines (Figure 2). These cell lines are anchorage-dependent, non-tumourigenic, and non-invasive and therefore provide a model for the study of specific genes in adenoma to carcinoma progression (Paraskeva *et al*, 1984, 1989; Brunton *et al*, 1997).

Fascin expression was not clearly detected under these conditions in BH/C1 and RG/C2, even though the blots shown represent a high level of exposure. However, the AA/C1 and AN/C1 adenoma cell lines showed readily detectable levels of fascin expression but much lower than those observed in the carcinoma-derived cell lines HT29 and SW480.

### Fascin expression increases motility in colorectal adenoma cells *in vitro*

Fascin expression in colorectal adenomas was found to relate to polyp size, histology and dysplasia in a manner consistent with a role in increasing malignant potential. Malignant transformation of tumours requires increased cell motility and we therefore used an *in vitro* model to determine whether fascin increases migration of benign colorectal adenoma cells.

**Figure 3:**
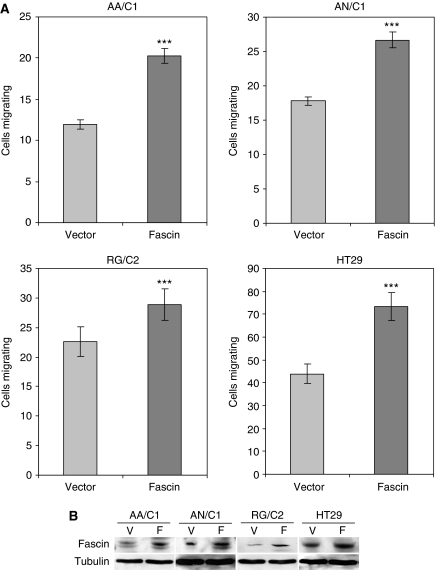
Fascin overexpression increases cell motility in both colorectal adenoma- and carcinoma-derived cell lines. (**A**) The forcible overexpression of fascin by transient transfection in adenoma- and carcinoma-derived cell lines increases cell motility *in vitro* (compared with vector-only control), as measured by Boyden chamber assay using 5% FBS as an attractant (^***^P<0.001 fascin transfected *vs* empty vector control). Three independent experiments were carried out in triplicate and the data are expressed as the mean±s.e.m. (**B**) Also shown are western blots performed to show the overexpression of fascin in the transfected cells. F=pCDNA3-fascin expression plasmid; V=vector control.

*In vitro* migration assays were carried out on three adenoma- and two carcinoma-derived cell lines 24 h after transient transfection with either fascin or an empty vector control (Figure 3A). We have previously shown that fascin overexpression stimulates motility in the SW1222 cell line (Jawhari *et al*, 2003). Western blots were carried out to show the efficacy of transfection (Figure 3B). Densitometric analysis of the western blots was performed and fold control increases of 2.36, 2.78, 4.04 and 1.55 for the AA/C1, AN/C1, RG/C2 and HT29 cell lines were observed, respectively.

A further 24 h after transfection, cell migration was significantly increased by fascin overexpression compared with vector controls in each of the adenoma cell lines tested (AA/C1, AN/C1 and RG/C2), with a comparative response in the carcinoma-derived cell line HT29.

## Discussion

Despite the continued broadening of our knowledge base, tumour spread results in 90% of all cancer deaths, and around half of patients presenting with colorectal cancer have advanced disease at clinical presentation (Young and Rea, 2000; Sporn, 1996). Therefore, there is a continuing need for new targets for disease management and a greater understanding of malignant progression required.

The dynamic rearrangements required in the cytoskeleton to allow cell motility and ergo tumour invasion require the action of multiple proteins including actin cross-linkers, such as fascin. The expression of fascin has been shown to be low or absent in the normal epithelium of a number of tissues, but deregulated in cancer (Hashimoto *et al*, 2005). We, and others, have recently shown that fascin expression is readily detected in the epithelial component of colorectal carcinomas (Jawhari *et al*, 2003; Hashimoto *et al*, 2006; Puppa *et al*, 2007). These data show that the expression of fascin represents a phenotypic change in colorectal carcinomas and that fascin is associated with a poorer prognosis in colorectal cancer patients (Hashimoto *et al*, 2006; Puppa *et al*, 2007). To understand fully the role of fascin in colorectal tumorigenesis, it is important to determine at what stage it becomes deregulated. Furthermore, no study has reported on the potential use of fascin as a biomarker for malignant progression in the earlier stages of colorectal tumourigenesis; for example, this type of marker could prove useful in determining the level of clinical follow-up required in patients following polypectomy. In this study, we sought to determine whether fascin has a role in malignant progression. Hashimoto *et al* (2006) found altered fascin expression in colorectal adenomas, but found no difference in fascin expression between FAP and sporadic tumours. In the study presented here, we have assessed a cohort of adenomas for fascin expression and correlated these data with the size, histology and dysplasia in the tumours. These three characteristics are known to be related to one another, but are also thought to be the crucial criteria for determining the malignant potential of an adenoma (Hamilton and Aaltonen, 2001). These factors have not previously been described in relation to fascin expression in colorectal adenomas.

Our data not only show a consistent overexpression of fascin in adenomas compared with normal tissue, but also significant positive correlations between the expression of fascin with tumour size, histology, dysplasia, both in terms of the proportion of the epithelium staining positively and the intensity of immunoreactivity. The strongest correlation was observed between fascin expression and the degree of dysplasia.

Analysis of fascin expression in adenoma-derived cell lines could also be considered to show the same relationship with adenoma size seen *in vivo*. If graded by the size of the original polyp from which they were derived, the BH/C1 (<0.5 cm), RG/C2 (1–2 cm), AN/C1 (1–2 cm) and AA/C1 (3–4 cm) cell lines (Paraskeva *et al*, 1984, 1989) show a direct correlation with the level of fascin expression.

The observation of fascin immunoreactivity focussed around the stalk of the polyps augments the idea that fascin could be important in malignant progression, as this is the potential site for malignant invasion. This localisation of fascin at the point of maximal contact with the underlying mesenchyme also allows us to speculate on the mechanism of fascin regulation, raising the possibility of epithelial–mesenchymal crosstalk directly affecting fascin expression. If true, this regulation may occur through alterations in the extracellular matrix deposited by the epithelium and the underlying mesenchyme, or through the secretion of paracrine factors affecting fascin transcription in the epithelial cells.

The functional consequence of fascin expression in adenomas was demonstrated through the use of *in vitro* cell motility assays. The adenoma cell lines represent benign tumours and are anchorage-dependent and non-tumorigenic. The role of fascin in the pre-malignant stages of colorectal tumorigenesis has not previously been studied *in vitro*. Fascin overexpression promoted cell motility in all the cell lines tested. The acquisition of motile behaviour is necessary for localised invasion and therefore malignancy. Therefore, the observed focal expression of fascin around the stalk of large adenomas *in vivo*, combined with the promotion of cell motility *in vitro*, implies that fascin overexpression may be important in the malignant progression of colorectal tumours.

## Figures and Tables

**Table 1:** Categorisation of colorectal adenoma samples (*n*=64) used for immunohistochemical study of fascin expression showing the number of specimens in each category

	**Histological type**		**Degree of dysplasia**		
**Adenoma size**	**Tubular**	**Tubulovillous**	**Mild**	**Moderate**	**Severe**
Small (<0.5 cm)	12	2	8	6	0
Medium (0.5–3.5 cm)	8	22	3	23	4
Large (>3.5 cm)	0	20	0	14	6

**Table 2:** Statistical correlation (Kendall's tau B) between fascin immunoreactivity and tumour size, histological type and degree of dysplasia in colorectal adenomas

	**Proportion of positive epithelial cells**			**Intensity of epithelial staining**				**Focal staining around stalk**	
**Adenoma characteristics**	**<20%**	**20–80%**	**>80%**	**0**	**1**	**2**	**3**	**Yes**	**No**
*Polyp size*									
S	13	1	0	8	3	2	1	3	11
M	22	5	3	7	6	2	15	20	10
L	12	6	2	2	1	6	11	15	5
TauB	0.254^*^			0.344^**^				0.344^**^	
			
*Histology*									
T	19	0	1	9	4	1	6	7	13
TV	28	12	4	9	3	8	24	31	13
TauB	0.288^*^			0.287^*^				0.335^**^	
			
*Dysplasia*									
Mild	10	0	1	7	1	1	2	1	10
Mod	35	9	2	9	7	9	21	33	13
Sev	3	3	1	0	0	1	6	6	1
TauB	0.244^*^			0.448^**^				0.437^**^	

Numbers given indicate the number of samples in each category and the Kendall's TauB correlation coefficient (between −1 and +1) The statistical significance of the correlation is indicated as follows: ^*^P=<0.05, ^**^P=<0.01.

Polyp size: small≤0.5 cm, medium0.5–3.5 cm, large≥3.5 cm; histology: tubular (T) or tubulovillous (TV); dysplasia: mild, moderate, severe.
